# Double major papilla of Vater - a rare endoscopic finding during endoscopic retrograde cholangiopancreatography: a case report

**DOI:** 10.1186/1757-1626-2-163

**Published:** 2009-10-21

**Authors:** Panagiotis Katsinelos, Grigoris Chatzimavroudis, Kostas Fasoulas, Taxiarchis Katsinelos, Ioannis Pilpilidis, Georgia Lazaraki, Sotiris Terzoudis, George Kokonis, Ioannis Patsis, Christos Zavos, Jannis Kountouras

**Affiliations:** 1 Department of Endoscopy and Motility Unit, "G Gennimatas" General Hospital, Thessaloniki, Greece; 2 Department of Gastroenterology, Second Medical Clinic, Aristotle University of Thessaloniki, Ippokration Hospital, Thessaloniki, Greece

## Abstract

**Background:**

A double major papilla of Vater is a rare congenital anomaly with only three documented cases described in the literature.

**Case report:**

We report the case of a 19-year-old man, with chronic ulcerative pancolitis and congenital sphrerocytosis, who underwent endoscopic retrograde cholangiopancreatography because he had persistent elevation of liver enzymes and normal MRI cholangiography. During endoscopic retrograde cholangiopancreatography, a double papilla of Vater with separate drainage for the bile duct and the pancreatic duct was observed.

**Conclusion:**

Endoscopic retrograde cholangiopancreatography showed normal pancreatogram and findings compatible with sclerosing cholangitis.

## Introduction

The Vaterian system includes the common bile duct and the main pancreatic duct (duct of Wirsurg), which, as they conjoin at the level of duodenum, they form the major papilla of Vater. Variations in the bile duct and pancreatic duct opening are related to the process of rotation and recanalization during embryologic development. Complete non-union of distal common bile duct and duct of Wirsurg gives rise to a double papilla of Vater [1-3].

We report a young man with double papilla of Vater wherein cannulation of the common bile duct and pancreatic duct could be achieved through either orifice independently.

## Case presentation

A 19-year-old Greek man was referred to our unit for endoscopic retrograde cholangiopancreatography (ERCP) with suspicion of sclerosing cholangitis, because of persistent elevated levels of asparate aminotransferase, alanine aminotransferase and alkaline phosphatase and normal MRI cholangiography.

Medical history revealed congenital spherocytosis and ulcerative pancolitis.

**Figure 1:**
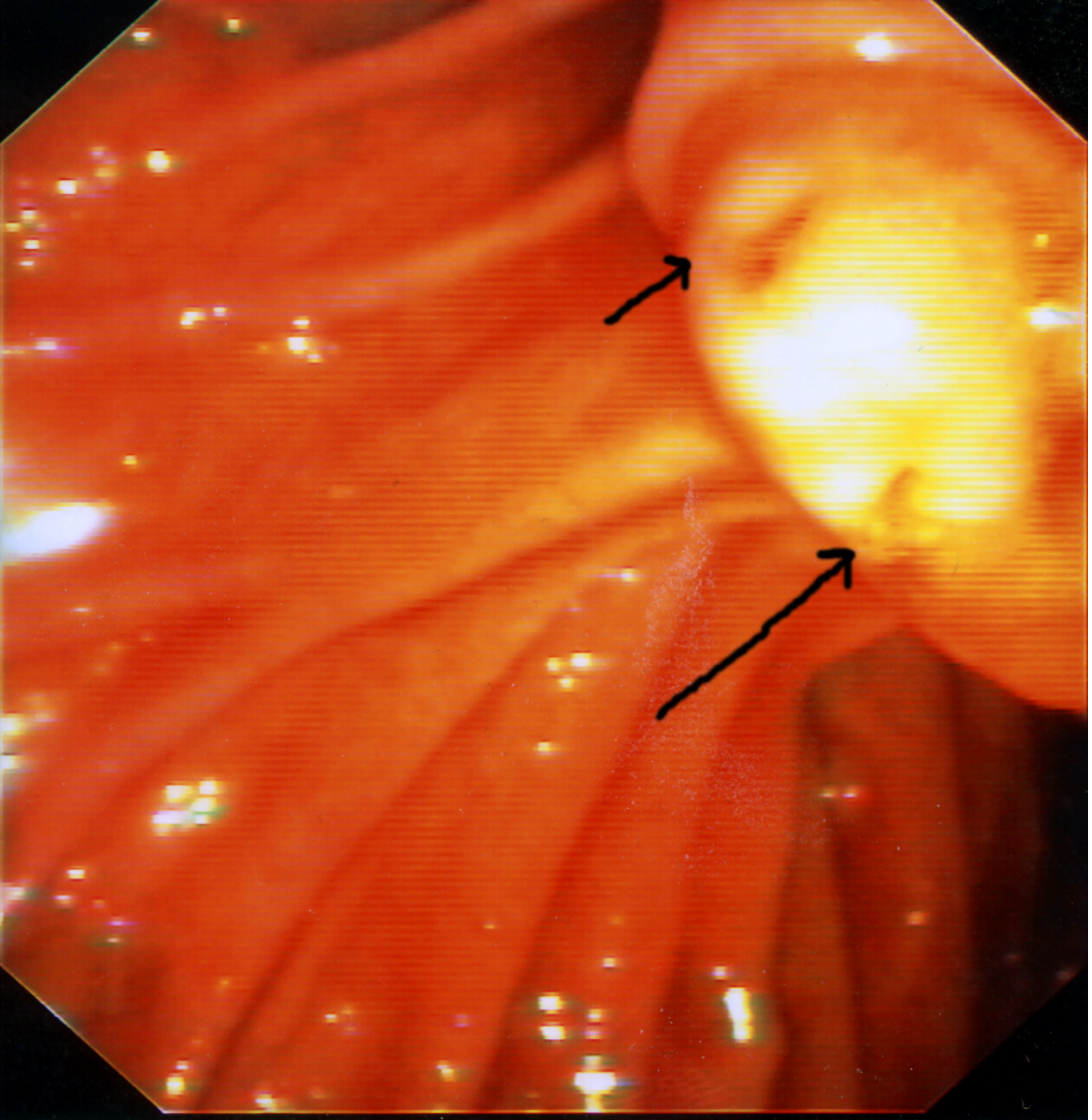
**Double major papilla**. The bile duct orifice is located superiorly, at an 11o'clock orientation (short arrow). The pancreatic duct orifice is located immediately inferiorly (long arrow), at a 5 o'clock orientation.

**Figure 2:**
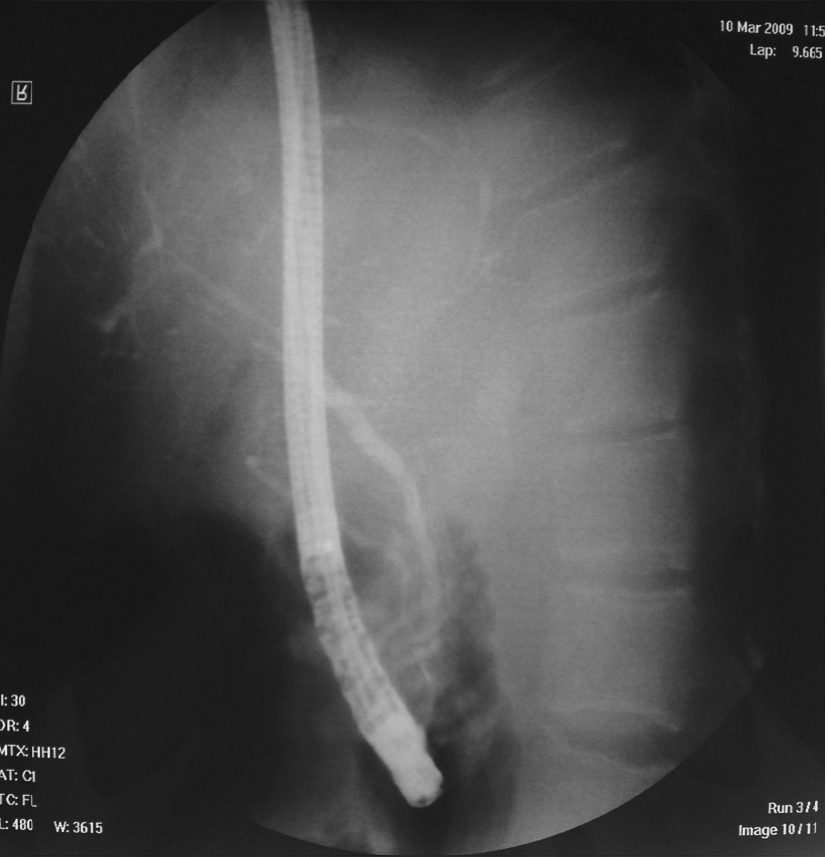
**Cholangiography showing intrabiliary stenoses and dilations, findings compatible with sclerosing cholangitis**. Pancreatography is normal.

At ERCP, inspection of the major papilla showed the presence of two separate orifices on a single papilla (Fig. 1). Each orifice demonstrated the "fronding" pattern (short and long arrow), often seen on the orifice of Vater's papilla. The cranial orifice was located at the 11 o'clock orientation, while the caudal orifice was positioned at the 5 o'clock orientation. During the endoscopic intervention, bile draining from the cranial orifice was noticed. Cannulation of this orifice led to visualization of extra and intrabiliary tract, a fact that proved that this orifice was the orifice of the common bile duct. The findings of the cholangiogram (intrabiliary stenoses and dilations) were compatible with sclerosing cholangitis (Fig. 2). Cannulation of the caudal orifice with injection of contrast showed normal pancreatogram (Fig. 2). We confirmed that there were two separate ampullary structures in the duodenal wall and not an acquired choledochoduodenal fistula because the catheter that was introduced through the caudal papilla did not come out through the cranial orifice. There were no complications related to the ERCP.

## Discussion

Interest in the anatomy of the descending duodenum and papilla began with advent of ERCP as diagnostic procedure in the early 1970s. The liver, gallbladder, biliary tree and pancreas originate in the hepatic diverticulum, which develops at the beginning of the 4^th ^embryonic week [3]. At this week, the developing extrahepatic biliary tree becomes organized. Establishment of the lumen of the extrahepatic biliary tree occurs by a process of recanalization [1]. During the recanalization process, the common bile duct and the pancreatic duct coalesce within one duct, the ampulla, before they open into the duodenum on the major papilla [1-3]. If they fail to coalesce, they remain as independent ducts and open into the duodenum separately [1-3].

Silvis et al described variations in the normal duodenal papilla at ERCP [4]. They commented that double papilla of Vater is a rare anatomic variant and is not usually apparent without close inspection. The existence of a double major papilla with two neighboring independent papillary structures which drain biliary and pancreatic ducts separately, is very unusual. Simon et al observed a double papilla of Vater in 0.18% of 1800 patients who underwent ERCP [5]. A search in Medline revealed only three documented cases of double papilla of Vater [5-7]. The existence of this rare anatomic anomaly does not predispose to any pancreatobiliary disease, but is an opportunity for another learning experience contributing to expert competency in ERCP and improved quality of care for patients.

Our case is intriguing because the patient had two independent papillary structures with two distinct, separate orifices and each orifice demonstrated prominent papillary fronds, typically seen at the ostium of the major papilla. Moreover there was no communication between the two orifices during cannulation with catheter, excluding the development of an acquired choledochoduodenal fistula.

## Conclusion

Our case demonstrates that if the endoscopist observes two separate papillae in the duodenum, with the proximal one draining the bile duct and the distal one draining the pancreatic duct, without communication between them, this depicts a rare anatomic variation.

## Consent

Written informed consent was obtained from the patient for publication of this case report and accompanying images. A copy of the written consent is available for review by the Editor-in-Chief of this journal.

## Competing interests

The authors declare that they have no competing interests.

## Authors' contributions

PK performed the endoscopy and was contributor in revising the manuscript critically for important intellectual content. GC was major contributor in writing the manuscript. KF, TK and IP analyzed and interpreted the patient data and were contributors in writing the manuscript. GL, ST, GK, and IP reviewed the relative literature. CZ and JK were major contributors in revising the manuscript critically for important intellectual content. All authors read and approved the final manuscript.
